# Twitter Conversations About Pancreatic Cancer by Health Care Providers and the General Public: Thematic Analysis

**DOI:** 10.2196/31388

**Published:** 2022-03-24

**Authors:** Udhayvir Singh Grewal, Arjun Gupta, Jamie Doggett, Emil Lou, Niraj J Gusani, Anirban Maitra, Muhammad Shaalan Beg, Allyson J Ocean

**Affiliations:** 1 Department of Internal Medicine Louisiana State University Health Sciences Center Shreveport, LA United States; 2 Sidney Kimmel Comprehensive Cancer Center Baltimore, MD United States; 3 Creation Maidstone United Kingdom; 4 Division of Hematology and Oncology Department of Medicine University of Minnesota Medical School Minneapolis, MN United States; 5 Baptist MD Anderson Cancer Center Jacksonville, FL United States; 6 Sheikh Ahmed Center for Pancreatic Cancer Research University of Texas MD Anderson Cancer Center Houston, TX United States; 7 Division of Hematology and Oncology Department of Medicine University of Texas Southwestern Medical Center Dallas, TX United States; 8 Weill Cornell Medicine New York Presbyterian Hospital New York, NY United States

**Keywords:** pancreatic cancer, Twitter, general public, health care providers, social media, Creation Pinpoint, thematic analysis

## Abstract

**Background:**

There is a growing interest in the pattern of consumption of health-related information on social media platforms.

**Objective:**

We evaluated the content of discussions around pancreatic cancer on Twitter to identify subtopics of greatest interest to health care providers and the general public.

**Methods:**

We used an online analytical tool (Creation Pinpoint) to quantify Twitter mentions (tweets and retweets) related to pancreatic cancer between January 2018 and December 2019. Keywords, hashtags, word combinations, and phrases were used to identify mentions. Health care provider profiles were identified using machine learning and then verified by a human analyst. Remaining user profiles were classified as belonging to the general public. Data from conversations were stratified qualitatively into 5 domains: (1) prevention, (2) survivorship, (3) treatment, (4) research, and (5) policy. We compared the themes of conversations initiated by health care providers and the general public and analyzed the impact of the Pancreatic Cancer Awareness Month and announcements by public figures of pancreatic cancer diagnoses on the overall volume of conversations.

**Results:**

Out of 1,258,028 mentions of pancreatic cancer, 313,668 unique mentions were classified into the 5 domains. We found that health care providers most commonly discussed pancreatic cancer research (10,640/27,031 mentions, 39.4%), while the general public most commonly discussed treatment (154,484/307,449 mentions, 50.2%). Health care providers were found to be more likely to initiate conversations related to research (odds ratio [OR] 1.75, 95% CI 1.70-1.79, *P*<.001) and prevention (OR 1.49, 95% CI 1.41-1.57, *P*<.001) whereas the general public took the lead in the domains of treatment (OR 1.63, 95% CI 1.58-1.69, *P*<.001) and survivorship (OR 1.17, 95% CI 1.13-1.21, *P*<.001). Pancreatic Cancer Awareness Month did not increase the number of mentions by health care providers in any of the 5 domains, but general public mentions increased temporarily in all domains except prevention and policy. Health care provider mentions did not increase with announcements by public figures of pancreatic cancer diagnoses. After Alex Trebek, host of the television show *Jeopardy*, received his diagnosis, general public mentions of survivorship increased, while Justice Ruth Bader Ginsburg’s diagnosis increased conversations on treatment.

**Conclusions:**

Health care provider conversations on Twitter are not aligned with the general public. Pancreatic Cancer Awareness Month temporarily increased general public conversations about treatment, research, and survivorship, but not prevention or policy. Future studies are needed to understand how conversations on social media platforms can be leveraged to increase health care awareness among the general public.

## Introduction

Social media platforms have emerged as tools for patients to access general health-related information and stay up-to-date with the latest therapeutic advancements [1,2]. Social media allows sharing information on cancer screening, prevention, treatment, and survivorship [3-6]. Apart from patients with cancer and their caregivers, cancer centers and patient advocacy groups use social media to disseminate content for patient education and fundraising activities [7]. There is a growing interest in the pattern and nature of the consumption of information by the general public through these platforms. Twitter is a micro-blogging website that can be used for sharing content with users around the world in real time. Tweets (short messages that are limited to a maximum of 280 characters) serve as a quick and efficient source of information that can then be liked, shared (retweeted) or commented on by other users to amplify and to maximize outreach on a common platform [8].

Pancreatic cancer is an intractable malignancy that is associated with a heavy burden of symptoms and poor overall survival [9]. Patients, caregivers, care teams, and researchers use Twitter as a platform to connect and share information related to pancreatic cancer treatments. It has also been used as a platform for advocating for needs and concerns that are unique to patients with pancreatic cancer [10]. However, there is a need to further analyze factors that drive these conversations and how they can be used as opportunities for initiating discussions on topics such as early detection, policy reforms, and survivorship. Additionally, several high-profile public figures have developed pancreatic cancer in recent years. Studying the impact of these events on the volume and nature of conversations can serve as a valuable case study in evaluating the influence of social media on cancer awareness.

We conducted the current analysis to study the themes and dynamics of conversations around pancreatic cancer on Twitter. We looked to study how health care providers and the general public use this platform. We also investigated the impact of Pancreatic Cancer Awareness Month and the diagnoses of public figures on conversations about pancreatic cancer.

## Methods

**Figure 1:**
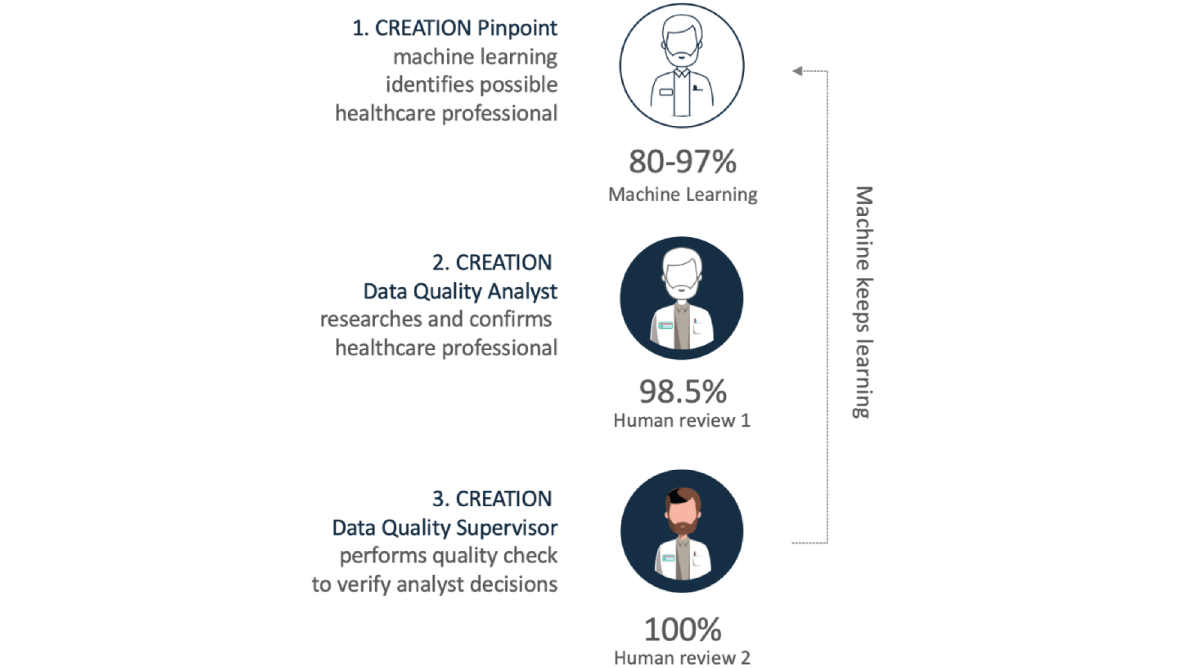
The analytical tool used in this study, Creation Pinpoint, uses machine learning algorithms to identify possible health care provider profiles on Twitter that are later confirmed and verified by data analysts.

We used an online analytical tool (Creation Pinpoint) to quantify Twitter mentions (tweets and retweets) related to pancreatic cancer made between January 2018 and December 2019. Keywords, hashtags, word combinations, and phrases were used to search for Twitter mentions related to pancreatic cancer. Perspectives from Twitter users were then distilled based on their online behaviors. Machine learning techniques were used to identify health care providers based on their Twitter profile description (commonly known as a Twitter bio). All health care provider profiles were then verified by a human analyst based on professional websites and other sources. Duplicate profiles or profiles that could not be verified were excluded. Only physicians were included as health care providers. In the final analysis, 13,788 health care providers were included. Analyst decisions were verified in a quality check performed by a data quality supervisor (Figure 1). All remaining user profiles were classified as belonging to the general public. After identification of tweets related to pancreatic cancer, data from conversations were analyzed and stratified qualitatively using keywords, combinations, and phrases into 5 domains (Table 1).

The month of November is Pancreatic Cancer Awareness Month. We analyzed the effect of Pancreatic Cancer Awareness Month in 2018 and 2019 on Twitter mentions in each of the 5 domains. Two prominent personalities announced a diagnosis of pancreatic cancer during the study period: Alex Trebek, host of the television show *Jeopardy*, in March 2018 and Justice Ruth Bader Ginsburg in August 2019. Additionally, Aretha Franklin passed away from pancreatic cancer in August 2018. We studied the effect of these 3 public figure cancer diagnoses on Twitter conversations initiated by health care providers and the general public in the domains described above.

**Table 1:** Search strategies for identification of tweets related to pancreatic cancer and further categorization into 5 domains: prevention, survivorship, treatment, research, and policy.

Search term	Keywords, combinations, and phrases
Pancreatic cancer	( pancchat OR pancan OR pancreaticcancer* OR pancreascancer* OR WorldPancreaticCancerDay OR #WPCD OR ((pancreatic OR Pancreas OR pancpath OR acinar OR vipoma OR somatostatinoma OR glucagonoma OR insulinoma OR gastrinoma OR pseudopapillary) AND (cancer OR adenocarcinoma OR carcinoma OR malignant OR tumor OR tumour)) OR ((PDAC OR PancNET OR PNET) AND (pancreatic OR Pancreas OR pancpath OR cancer OR tumor OR tumour OR mutated OR metastatic)) OR ((pancreatic OR Pancreas OR pancpath OR acinar OR vipoma OR somatostatinoma OR glucagonoma OR insulinoma OR gastrinoma OR pseudopapillary OR PDAC OR PancNET OR PNET) AND (gemcitabine OR paclitaxel OR FOLFIRI OR mFOLFIRI OR FOLFOX OR fluorouracil OR 5FU OR irintecan OR irinotecan OR everolimus OR evorolimus OR oxaliplatin OR cisplatin OR “demplatin pegraglumer” OR capacitabine OR capecitabine OR docetaxel OR carboplatin OR glufosfamide OR glucosphamide OR leucovorin OR folinic OR tetrahydrofolic OR pembralizumab OR pembrolizumab OR nivolumab OR ipulimumab OR ipalimumab OR ipilimumab OR ipilumab OR cabiralizumab OR cabaralizumab OR cabarilizumab OR urelumab OR olaratumab OR talabostat OR cobimetanib OR cobimetinib OR anetumab OR epacadostat OR atezolizumab OR pamrevlumab OR pegilodecakin OR PEGylated OR PEGPH20 OR pegvorhyaluronidase OR avelumab OR bempegaldesleukin OR erlotinib OR sunitinib OR olaparib OR rucaparib OR napabucasin OR masican OR masitinib OR velaparib OR veliparib OR GVAX OR CRS207 OR Gemzar OR infugem OR onxol OR taxol OR abraxane OR adrucil OR efudex OR efudix OR carac OR onivyde OR campto OR afinitor OR zortress OR eloxatin OR platinol)) ) AND site:twitter.com
Prevention	prevent* OR screen OR screening OR ((reduce OR decrease OR lower OR limit) NEAR/3 (risk))
Survivorship	survivor* OR survival OR “OS” OR “PFS” OR overcome OR beat
Treatment	treat OR treatment* OR treating OR gemcitabine OR paclitaxel OR “nab-paclitaxel” OR FOLFIRI* OR mFOLFIRI*,…….
Research	Research OR study OR trial OR trials OR studies OR data
Policy	policy OR policymak* OR ((NIH or NCI)) AND (fund*)) OR (insurance AND expan*)

## Results

### Classification by Domain

**Figure 2:**
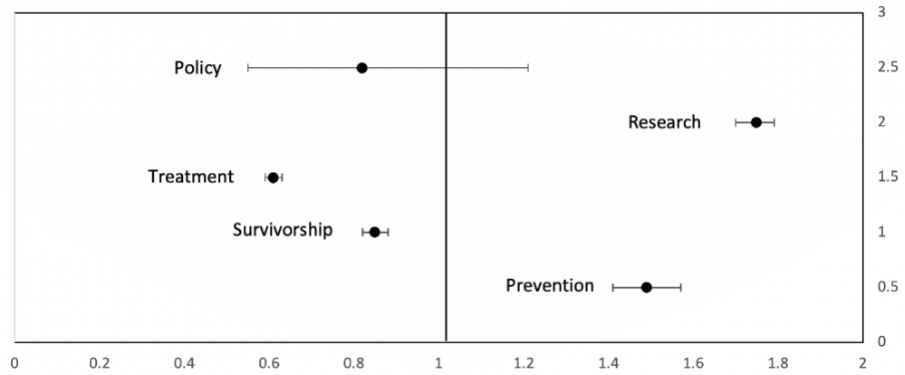
Forest plot depicting the odds ratio for conversations related to pancreatic cancer initiated by health care providers and the general public in the domains of policy, research, treatment, survivorship, and prevention.

We identified a total of 1,258,028 English-language mentions related to pancreatic cancer from January 2018 to December 2019, out of which 62,439 were from health care providers and 1,195,598 were from the general public. Out of 1,258,028 mentions, we identified a total of 313,668 unique mentions (27,031 by health care providers and 307,449 by the general public) that were classified into the 5 domains of prevention, treatment, research, survivorship, and policy. Health care providers most often discussed pancreatic cancer research (10,640/27,031 mentions, 39.4%) while the general public most often discussed treatment (154,484/307,449 mentions, 50.2%). Health care providers focused the least on policy (28/27,031 mentions, 0.1%); the general public also focused the least on policy (93/27,031 mentions, 3.3%). A comparative analysis showed that health care providers were more likely to initiate conversations related to research (odds ratio [OR] 1.75, 95% CI 1.70-1.79, *P*<.001) and prevention (OR 1.49, 95% CI 1.41-1.57, *P*<.001) whereas the general public took the lead in the domains of treatment (OR 1.63, 95% CI 1.58-1.69, *P*<.001) and survivorship (OR 1.17, 95% CI 1.13-1.21, *P*<.001). As shown in Figure 2, health care providers were not found to be more likely to initiate conversations in the domain of policy when compared to the general public (OR 0.82, 95% CI 0.55-1.21, *P*=.32). The temporal distribution of mentions in each category for both health care providers and the general public is shown in Table 2.

**Table 2:** Stratification of Twitter mentions by health care providers and the general public from January 2018 to December 2019.

Month, year	Prevention, n (%)			Survivorship, n (%)			Treatment, n (%)			Research, n (%)			Policy, n (%)	
	HCP^a^	GP^b^	HCP		GP	HCP		GP	HCP		GP	HCP		GP
Jan 2018	62 (8.3)	620 (10.2)	132 (5.9)		1276 (4.6)	435 (9.5)		3409 (6.7)	405 (8.3)		4005 (10.0)	0 (0)		2 (0.8)
Feb 2018	89 (11.9)	648 (10.6)	97 (4.4)		967 (3.5)	184 (4.0)		2076 (4.1)	314 (6.4)		2907 (7.3)	0 (0)		2 (0.8)
Mar 2018	76 (10.2)	547 (8.9)	98 (4.4)		2805 (10.1)	347 (7.6)		3498 (6.9)	372 (7.6)		3151 (7.9)	2 (18.2)		37 (14.2)
April 2018	69 (9.2)	377 (6.2)	115 (5.2)		1026 (3.7)	285 (6.2)		2050 (4.0)	410 (8.4)		2521 (6.3)	1 (9.1)		16 (6.2)
May 2018	65 (8.7)	649 (10.7)	158 (7.1)		1576 (5.7)	438 (9.6)		4841 (9.5)	424 (8.7)		3162 (7.9)	1 (9.1)		17 (6.5)
June 2018	54 (7.3)	286 (4.7)	531 (23.9)		3510 (12.6)	903 (19.7)		5092 (10.0)	854 (17.5)		6322 (15.8)	1 (9.1)		12 (4.6)
July 2018	35 (4.7)	248 (4.1)	95 (4.3)		1073 (3.9)	244 (5.3)		2811 (5.5)	255 (5.2)		2629 (6.6)	0 (0)		3 (1.2)
Aug 2018	47 (6.3)	634 (10.4)	137 (6.2)		3360 (12.1)	244 (5.3)		4184 (8.2)	380 (7.8)		3285 (8.2)	1 (9.1)		6 (2.3)
Sept 2018	36 (4.8)	250 (4.1)	122 (5.5)		1046 (3.8)	246 (5.4)		2441 (4.8)	392 (8.0)		2780 (6.9)	0 (0)		1 (0.4)
Oct 2018	80 (10.7)	833 (13.7)	114 (5.1)		1242 (4.5)	231 (5.0)		3816 (7.5)	287 (5.9)		2497 (6.2)	1 (9.1)		108 (41.5)
Nov 2018	106 (14.2)	802 (13.2)	297 (13.4)		4137 (14.9)	552 (12.04)		13,428 (26.5)	460 (9.4)		4907 (12.3)	3 (27.3)		52 (20.0)
Dec 2018	29 (3.9)	201 (3.3)	321 (14.5)		5796 (20.8)	472 (10.3)		3106 (6.1)	331 (6.8)		1793 (4.5)	1 (9.1)		4 (1.5)
Jan 2019	60 (6.2)	390 (4.7)	178 (7.8)		1350 (3.9)	337 (3.0)		3365 (3.0)	506 (8.8)		2844 (5.8)	0 (0)		1 (0.4)
Feb 2019	74 (7.6)	534 (6.4)	124 (5.4)		1543 (4.5)	212 (3.8)		3200 (2.9)	357 (6.2)		3109 (6.4)	0 (0)		6 (2.4)
Mar 2019	83 (8.5)	692 (8.3)	271 (11.9)		10,724 (31.4)	595 (10.7)		5930 (5.3)	605 (10.5)		5195 (10.6)	0 (0)		28 (11.4)
April 2019	56 (5.8)	310 (3.7)	165 (7.2)		1097 (3.2)	522 (9.4)		3865 (3.5)	525 (9.1)		3911 (8.0)	2 (11.8)		27 (10.9)
May 2019	116 (11.9)	902 (10.8)	171 (7.5)		1965 (5.7)	565 (10.2)		9929 (8.9)	503 (8.7)		3169 (6.5)	2 (11.8)		18 (7.3)
June 2019	42 (4.3)	424 (5.1)	299 (13.1)		1693 (4.9)	772 (13.9)		4589 (4.1)	686 (11.9)		4861 (9.9)	0 (0)		12 (4.9)
July 2019	40 (4.1)	1965 (23.6)	144 (6.3)		795 (2.3)	428 (7.7)		3782 (3.4)	444 (7.7)		2948 (6.0)	4 (23.5)		25 (10.2)
Aug 2019	176 (18.1)	941 (11.3)	221 (9.7)		3842 (11.2)	562 (10.1)		36,267 (32.6)	388 (6.7)		4635 (9.5)	6 (35.3)		21 (8.5)
Sept 2019	65 (6.7)	333 (3.9)	193 (8.5)		1759 (5.1)	272 (4.9)		6813 (6.1)	452 (7.9)		3580 (7.3)	1 (5.9)		21 (8.5)
Oct 2019	105 (10.8)	599 (7.2)	131 (5.7)		1684 (4.9)	299 (5.4)		5529 (4.9)	390 (6.8)		3324 (6.8)	0 (0)		20 (8.1)
Nov 2019	99 (10.2)	674 (8.1)	280 (12.3)		5405 (15.8)	456 (8.2)		7385 (6.6)	644 (11.2)		8206 (16.8)	2 (11.8)		50 (20.3)
Dec 2019	55 (6.7)	578 (6.9)	105 (4.6)		2318 (6.8)	544 (9.8)		20,672 (18.6)	256 (4.5)		3048 (6.2)	0 (0)		17 (6.9)
Total - 2018, n	748	6095	2217		27,814	4581		50,752	4884		39,959	11		260
Total - 2019, n	971	8342	2282		34,175	5564		111,326	5756		48,830	17		246

^a^HCP: health care provider.

^b^GP: general public.

### Impact of Pancreatic Cancer Awareness Month

**Figure 3:**
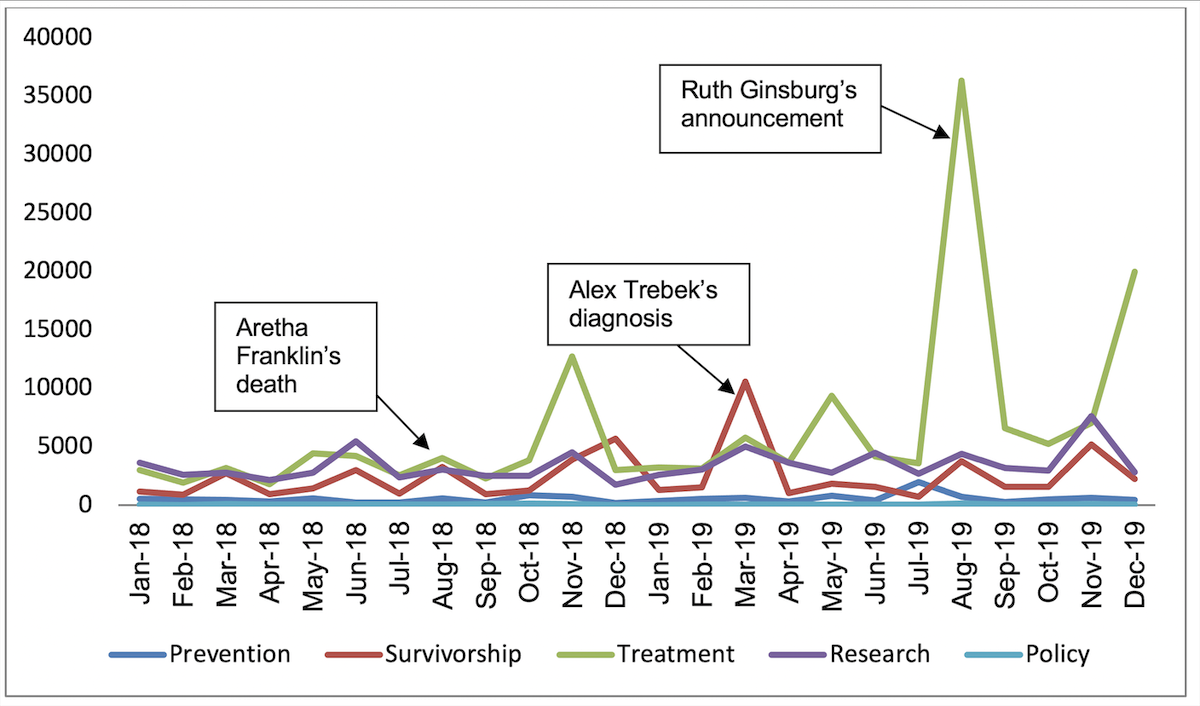
Impact of announcements by public figures of pancreatic cancer diagnoses on Twitter mentions related to pancreatic cancer.

Pancreatic Cancer Awareness Month did not increase pancreatic cancer mentions by health care providers in any of the 5 domains. However, over the study period of 2 years, mentions by the general public increased for treatment, survivorship, and research. Mentions of the topics of prevention and policy did not increase during Pancreatic Cancer Awareness Month (Figure 3).

### Impact of Announcements by Public Figure of Pancreatic Cancer Diagnoses

We analyzed the impact of announcements by public figures of pancreatic cancer diagnoses on Twitter conversations. Conversations initiated by health care providers did not change with announcements by public figures of pancreatic cancer diagnoses. Among the general public, Mr Trebek’s diagnosis was associated with increased conversations about survivorship and Justice Ginsburg’s diagnosis was associated with increased conversations about treatment (Figure 3). The announcement of Ms Franklin’s death did not result in changes in any of the 5 domains studied as a part of the analysis.

## Discussion

### Principal Findings

We analyzed Twitter conversations about pancreatic cancer between 2018 and 2019. Twitter discussions by health care providers did not align with discussions initiated by the general public. Pancreatic Cancer Awareness Month did not increase conversations in any of the 5 domains for health care providers, but general public conversations increased in all domains except prevention and policy. Pancreatic cancer announcements by public figures did not affect conversations initiated by health care providers and had varied impact on general public conversations. Mr Trebek’s diagnosis increased conversations about survivorship while Justice Ginsberg’s announcement increased conversations about treatment.

The current analysis highlights the importance of using social media platforms such as Twitter for analyzing the areas of greatest interest to health care providers and the general public in relation to cancer. The increased interest among the general public in pancreatic cancer treatment could be driven by the low survival rates of patients with pancreatic cancer. Pancreatic cancer is an aggressive malignancy; only about 15% to 20% of patients are diagnosed at an early stage and can benefit from potentially curative resection [11]. Despite advances in recent years, pancreatic cancer treatment continues to remain a formidable challenge. Our findings are in line with other studies that have highlighted the inclination of the general public toward cancer treatment–related discussions on Twitter. A pattern-matched analysis of cancer patients’ sentiments on Twitter revealed that patients were most likely to discuss their treatment course (ie, chemotherapy, radiation, and hospital visits). This analysis also identified pancreatic cancer as one of the cancer types associated with the lowest average happiness values among patients [3]. An analysis of Twitter conversations about lung cancer also revealed that users were most likely to tweet about treatment options, which included sharing their personal experiences with treatment or promoting information about newer therapies for lung cancer [12].

The “Twittersphere” also helps in building a communicative and collaborative atmosphere that allows health care providers to involve patients in their care by sharing the latest research and developments in the field [13]. Content experts and researchers can share their work and obtain feedback from the scientific community, patient advocacy groups, and the general public in real time [14]. Live Twitter chats are a unique way for those interested in pancreatic cancer to come together and discuss various topics, including research, policy, and treatment. #PancChat is a Twitter chat that was developed for discussion of relevant information related to pancreatic cancer treatment, diagnosis, and ongoing research with the pancreatic cancer community in a timely manner. #PancChat was developed in 2016 by the Let’s Win! Pancreatic Cancer Foundation [15] in collaboration with advocacy organizations and a pharmaceutical company. The organizers of the chat develop a series of questions based on the topic being discussed. The event is promoted through various social media platforms and at the time of the chat these questions are serially released. The ensuing conversations can be tracked using the #PancChat hashtag and can be catalogued for future reference. Approximately 20% of the users of #PancChat are patients, advocates, and non–health-care-related individuals. This suggests Twitter can be a powerful tool to disseminate health care information to health care providers, patients, and caregivers [10].

Cancer awareness months are focused on increasing recognition of the disease. Through our analysis, we studied the impact of Pancreatic Cancer Awareness Month on Twitter conversations. We found that Pancreatic Cancer Awareness Month increased conversations initiated by the general public, but that the increase was not uniform from year to year. There was no detectable difference in the domains of prevention and policy. The search algorithm used by our study included both primary prevention and early identification of pancreatic cancer in the prevention domain. There is a growing concern that early detection of pancreatic cancer does not receive adequate attention [16]. A study of Twitter conversations during Breast Cancer Awareness Month found that a majority of the tweets did not prioritize prevention or screening [17]. This suggests that stakeholders should ensure that conversations during Pancreatic Cancer Awareness Month consistently cover various attributes of pancreatic cancer care, including preventative measures. Targeted tweets and conversations specifically related to pancreatic cancer may be essential in increasing discussions on cancer prevention and early identification [10]. The use of machine learning to understand the content and dynamics of conversations related to pancreatic cancer on Twitter will allow the identification of gaps in awareness and communication among health care providers and the general public. This information can then be leveraged to design interventions to address deficiencies and improve communication in those specific areas in a focused manner. This knowledge will also add to the efficiency of targeted interventions such as tailored messaging, which may be used by health care organizations and advocacy groups to further augment dialogue around pancreatic cancer.

Public figure cancer diagnoses have been known to influence public behavior related to cancer. President Ronald Reagan’s diagnosis of colon cancer resulted in an increase in the number of colonoscopies performed on asymptomatic individuals [18]. Angelina Jolie’s op-ed in the *New York Times* regarding her risk-reducing bilateral mastectomy led to an increase in breast surgery among high-risk women [19]. In the current analysis, we found that a public figure being diagnosed with pancreatic cancer had different impacts on pancreatic cancer–related conversations initiated by the general public, depending on the public figure’s personal messaging around the diagnosis and the messaging of reports in the mainstream media. Our findings highlight that public figure diagnoses of pancreatic cancer offer a unique opportunity to capitalize on the increased attention of the general public to the disease. It has also been suggested that public figure cancer announcements can be used to augment conversations about prevention and early diagnosis of cancer [20]. There is a need to study in detail how public figure cancer diagnoses and deaths impact the content and dynamics of Twitter conversations. These data can help physicians, health care systems, and advocacy organizations engage in active communication with targeted audiences and encourage preventative behaviors on a large scale.

### Limitations

Limitations of the current study include a short study period and inclusion of tweets or mentions in English only. We did not study regional differences in discussion type. All users not identified as health care providers were identified as the general public, but a more detailed classification of non–health-care providers into patients, survivors, family and friends, advocacy groups, and professional organizations might lead to a better understanding of the conversations initiated by each of these groups. As well, granular details of the conversations could not be harvested or incorporated into the current analysis. Future studies that include a detailed sentiment analysis of the tweets in each domain would allow more insight into the nature and dynamics of Twitter conversations initiated by both health care providers and the general public. Various social media platforms are popular among different groups of users, which means that Twitter users are not representative of the general public. Twitter users are likely to be younger, wealthier, and more educated than the general public [21]. This analysis provides a framework that can be replicated across other social media platforms to gain insight into the conversations taking place about cancer.

### Conclusions

This study shows that Twitter conversations initiated by health care providers and the general public are not aligned. Health care providers focus most often on research, while treatment is the most popular topic among the general public. A better understanding of particular areas of interest to the general public might provide researchers, advocacy organizations, and health care systems the opportunity to identify unmet needs related to pancreatic cancer. Pancreatic Cancer Awareness Month increases general public conversations in multiple domains. There is a need to identify and implement strategies to use Pancreatic Cancer Awareness Month to stimulate dialogue that focuses on early detection of pancreatic cancer. Public figure diagnoses or deaths from pancreatic cancer can impact conversations related to pancreatic cancer among the general public. Future studies should also investigate factors that determine how public figure diagnoses impact conversations related to pancreatic cancer.

## Abbreviations

OR: odds ratio
